# International Experience of Damages Compensation in Armed Conflicts: Lessons for Ukraine

**DOI:** 10.12688/f1000research.171894.1

**Published:** 2025-11-13

**Authors:** Yuliia Hartman

**Affiliations:** 1 Department of Justice, Taras Shevchenko National University of Kyiv, Kyiv, Ukraine

**Keywords:** access to justice, right to compensation, compensation commissions, conflict-related claims, Ukraine war damages compensation, quasi-judicial mechanisms.

## Abstract

**Background:**

Access to justice, enshrined as a fundamental human right in international conventions, includes the right to a fair trial and to just compensation. This dimension of justice is particularly crucial in contexts of armed conflict, where victims of military aggression require effective reparation mechanisms. Historically, both judicial and quasi-judicial bodies have been created to address mass claims, from restitution in Bosnia and Herzegovina, Kosovo, and Kuwait to processes in post-authoritarian or post-communist states. Such mechanisms highlight the need for specialised institutions, as ordinary courts are often unable to manage the volume and complexity of conflict-related claims. Since Russia’s full-scale invasion of Ukraine in 2022, reparations have become central to legal and political debate.

**Methods:**

This study employed an empirical approach, combining systematic data collection with comparative legal analysis. Sources included UN and Council of Europe acts, reports by international organisations (CoE, OSCE), NGO publications, and scholarly works. Three compensation models were examined in depth: the UN Compensation Commission, Kosovo property claims mechanisms, and Bosnia’s Commission for Real Property Claims. These were selected for their effectiveness, European relevance, and addressed harms. Comparative analysis evaluated their procedures, accessibility, and recognition of harm, contrasted with Ukraine’s emerging compensation mechanism.

**Results:**

The study highlights lessons from the UNCC, Kosovo, and Bosnia, focusing on mandates, procedures, accessibility, and types of harm recognised. It identifies best practices and challenges, offering comparative insights for designing Ukraine’s future compensation system.

**Conclusions:**

International commissions share key features: defined jurisdiction, politically sensitive and lengthy processes, and reliance on transparent procedures to ensure legitimacy. For Ukraine, enabling direct individual claims is essential to uphold the right to reparation. Yet enforcement challenges and risks of duplicative proceedings remain. Adapting global experience to Ukraine’s context is crucial for developing an effective and trusted compensation mechanism.

## 1. Introduction

Everything begins with access to justice. Access to justice is one of the fundamental human rights, as affirmed by numerous international conventions. The right to a fair trial is multifaceted and encompasses an almost limitless range of interpretations of what may constitute fairness. In many instances, it does not even take the form of a trial in the classical sense. The right to reparation or compensation also falls within the scope of the right to a fair trial. Particular importance is attached to it in cases where the involvement of a competent authority is required to adjudicate claims for just compensation. Among the categories of cases in which it is impossible to proceed without such a body, one of the most prominent is compensation for harm caused by armed conflicts, military aggression or war.

Legal history provides numerous examples of both permanent institutions (courts) and specially established bodies (special commissions and tribunals) tasked with handling claims submitted by victims and adjudicating compensation issues. In recent decades, restitution rights have been recognised, and laws and procedures developed and enforced in various contexts: in post-conflict states such as Bosnia and Herzegovina, Georgia, and Kosovo; in post-authoritarian societies like South Africa and Iraq; and in post-communist countries. Quasi-judicial and administrative bodies have often been established to facilitate the timely resolution of conflict-related property claims, as the volume of such claims would overwhelm ordinary courts.

With the onset of Russia’s full-scale invasion of Ukraine in 2022, Ukrainian legal scholars and practitioners have focused their attention on the problem of compensating damages caused by Russia’s aggression. Debates continue regarding the possible mechanisms of reparation and the prospects for their practical enforcement. Drawing on international experience, the logical step would be the creation of a quasi-judicial body mandated to adjudicate cases related exclusively to compensation for damage inflicted by Russia’s war of aggression. As of early 2025, the primary avenue for realising the right to reparation is the international mechanism proposed by the Council of Europe, consisting of a Register of Damage, a Compensation Commission, and a Fund to be replenished with resources confiscated from both Russian sovereign and private assets. However, as is often said, the devil is in the detail. At present, the international compensation mechanism is still in its initial stage, focused on the collection of claims from individuals seeking compensation. The road ahead involves numerous stages before the right to a fair trial in this context can be fully realised.

To anticipate its effectiveness and outcomes, and to identify gaps and opportunities for improvement, it is essential to study the experience of other compensation commissions, as such mechanisms are not unprecedented. This study therefore aims to review and analyse several compensation bodies, including the United Nations Compensation Commission (UNCC), established by the UN Security Council following the 1990–1991 Gulf War to address, among other things, environmental damage caused by Iraq’s military invasion of Kuwait; the conflict-related mass-claims mechanisms in Kosovo; and the Commission for Real Property Claims in Bosnia, created under the Dayton Agreement to certify property rights for the many refugees and displaced persons of the Bosnian war.

The analysis will examine the establishment of these bodies, their procedural frameworks, and, in particular, their accessibility to victims and survivors seeking just compensation. Their operations will not only be studied but also assessed through the lens of the Ukrainian context and the needs of Ukrainian society. Special attention will be devoted to the types of harm recognised for compensation by each of these commissions. Comparing the categories of harm that have already been addressed in global practice will help illuminate the potential pathways for resolving compensation issues in Ukraine.

The conclusion will summarise the key lessons that Ukraine must learn to implement an effective compensation system for war victims and will propose recommendations for the design of Ukraine’s own compensation mechanism, taking into account the international experience reviewed.

## 2. Methods

In the course of this study, an empirical method of data collection was employed, which consisted of identifying and selecting relevant sources for subsequent analysis. The research examined a range of normative legal acts of the United Nations and the Council of Europe, reports issued by the Council of Europe, the OSCE, and international non-governmental organisations, as well as scholarly articles addressing the issue of reparations for harm caused by armed conflicts and aggression, and the operation of compensation commissions.

For detailed analysis, several models of compensation commissions were selected: the United Nations Compensation Commission (UNCC), the property claims commissions in Kosovo, and the commissions addressing displacement in Bosnia and Herzegovina. These models were chosen for the following reasons. The UNCC is considered one of the most successful examples of an international compensation mechanism administered under the auspices of the United Nations. Its experience is therefore valuable for comparison with the work of the international compensation mechanism for Ukraine, which operates under the aegis of another supranational institution—the Council of Europe. The commissions in Kosovo and Bosnia and Herzegovina represent unique European examples of reparation processes. Their experience is particularly relevant given the specific types of harm they addressed—types of harm that also require reparation in the context of the war in Ukraine.

In addition, a comparative analysis method was applied to contrast selected aspects of the functioning of these commissions with the emerging work of the compensation mechanism for Ukraine, as well as with the forms of reparation currently available to victims of the armed aggression of the Russian Federation against Ukraine.

## 3. International experience in the functioning of compensation commissions

### 3.1 United nations compensation commission

One of the most well-known precedents for the establishment and operation of a compensation commission is the UN-established body created to process compensation claims arising from the 1990–1991 Gulf War. It is widely regarded as a successful example, as it remains one of the few institutional mechanisms to date that has held a state accountable for its actions during armed conflict—particularly for the environmental damage resulting from those actions ( Cusato, 2019). It should be noted that the primary focus of this commission was on compensating damage specifically caused to the environment, highlighting the distinct nature not only of the subject matter of the claims but also of the parties involved in this ad hoc process.

The decision to invade Kuwait constituted a clear violation of the international order established by the UN Charter (1945), directly contravening the prohibition on the use of force set forth in Article 2(4). It is striking how history continually repeats itself, and the challenges faced by the United Nations are by no means new. At the same time, the willingness to address these challenges decisively and in accordance with the principles enshrined in the UN Charter remains questionable. In response to Iraq’s violation of international law and Article 2 of the UN Charter, a coalition of states was authorised to take countermeasures and use force.

As a result of Iraq’s armed invasion and its unlawful attempt to annex Kuwait, substantial environmental damage occurred, caused both by the aggressor and by the coalition forces acting to end the conflict. In particular, there was extensive use of mines and booby traps by both the Coalition Forces and Iraq. Evidence demonstrating the environmental impact of this military campaign was collected by the United Nations Environment Programme ( UNEP, 1993).

Following the resolution of the conflict, the UN Security Council adopted Resolution 687 (1991), which, in paragraph 16, held Iraq responsible for any direct losses or damage, including environmental harm and depletion of natural resources, as well as damage caused to foreign governments, citizens, or corporations arising from Iraq’s invasion of Kuwait. Pursuant to this resolution, the Security Council decided to establish a fund for the payment of compensation claims related to the damages identified in paragraph 16 and to create a commission responsible for reviewing claims and administering the fund.

In addition, it is important to note that the Secretary-General was tasked with developing recommendations for the fund’s implementation of compensation payments to claimants. These recommendations were to address the management of the compensation fund, mechanisms for determining Iraq’s appropriate contributions, measures to ensure timely payments into the fund, procedures for the allocation of funds and disbursement of compensation, methodologies for assessing damages, procedures for reviewing claims and verifying their validity, as well as resolving disputes regarding the liability of Iraq and the coalition states. The composition of the future Compensation Commission was also determined in accordance with these recommendations ( UN Security Council Resolution 687(1991), 1991).

In the case of Russia’s war of aggression against Ukraine, the UN General Assembly, in its resolution of 14 November 2022, recognised the need to establish, in cooperation with Ukraine, an international mechanism for the compensation of damage, losses, or other harm resulting from the unlawful actions of the Russian Federation against Ukraine or within Ukrainian territory. The resolution also affirmed that Russia is obligated to provide reparations in the future for the harm caused by these actions ( UNGA Resolution ES-11/5, 2022). However, no further steps were taken by the UN toward establishing a Compensation Commission for Ukraine. This responsibility was instead assigned to Member States in cooperation with Ukraine. Thus, the General Assembly effectively removed the future Compensation Commission for reparations related to Russia’s armed aggression against Ukraine from the UN’s direct competence and influence, abstaining from direct involvement in its establishment or operation. This decision was likely driven primarily by the fact that, had the Commission been created within the UN framework following the model of the UNCC for Kuwait, Russia could have employed all possible measures—including its veto power, the most potent tool capable of blocking any Security Council decision—to hinder the Commission’s full functionality.

In the case of Iraq, determining the state’s actual contribution to the compensation fund involved considering the value of Iraq’s oil and petroleum exports, the claims of the Iraqi people, Iraq’s capacity to pay (assessed jointly with international financial institutions, taking into account external debt servicing), and the needs of the Iraqi economy.

It is also important to highlight the deadlines set for preparing recommendations. According to paragraph 19 of Resolution 687, the Secretary-General was required to submit these recommendations no later than 30 days after the adoption of the Resolution. This underscores the Security Council’s prompt action in addressing the Iraq-Kuwait conflict and its recognition of reparations as a key component in restoring just peace. Legally, Resolution 687 laid a solid foundation for the creation and subsequent functioning of the Compensation Commission, as well as establishing the legal basis for holding Iraq accountable and ensuring fulfilment of its obligation to compensate for damages caused by the invasion and occupation of Kuwait.

It should be noted that the UN Compensation Commission was characterised by innovative features. With its creation, environmental damage and the depletion of natural resources were recognised for the first time in the context of war reparations, and a specific procedure for compensation was established.

Particular attention should also be given to the legal nature of the UN Compensation Commission, which handled claims for damages caused by Iraq’s invasion of Kuwait. As a general principle, international claims and compensation commissions are bodies established through agreements between two or more states, or by an international organisation, to resolve interstate claims—or private claims against a respondent state—most often arising from armed conflicts, internal unrest, or revolutionary events ( Romano, 2011). Some scholars even consider international compensation commissions as a form of arbitration institution ( Cusato, 2019). However, the UN Compensation Commission (UNCC) diverged from these general definitions by being granted exceptional powers. One of the main distinctions of the Commission from traditional arbitration was its departure from the “golden rule” that each party to a dispute appoints its own arbitrator. In this case, Iraq was deprived of the right to have its representatives participate in the Commission’s bodies.

The UN Compensation Commission (UNCC) operated under the direction of a 15-member Governing Council, composed of representatives from the current members of the UN Security Council. The Council was responsible for setting guidelines on essential matters, including the categorisation of claims, the definition of “direct loss,” submission requirements, and procedures for dispute resolution, as well as for making final determinations on all submitted claims. Assisting the Council was a group of commissioners—experts in law, finance, accounting, and environmental damage assessment—nominated by the UN Secretary-General and appointed by the Governing Council. Organised into three-member panels according to claim categories, the commissioners evaluated the evidence provided by claimants, verified the validity of claims, calculated the monetary value of losses, and submitted recommendations on compensation to the Governing Council.

As previously noted, Iraqi citizens and official representatives were not admitted to the Governing Council or the panels, which is entirely justified. Iraq, as the internationally recognised aggressor, bore the unquestionable obligation to remedy the consequences of its own aggression and restore justice—one key component of which was the payment of reparations and compensation for the damage caused. There could be no question of advocating for Iraq’s interests during the review of claims related to damage caused by its invasion and occupation. This approach aligns with the UN Guiding Principles developed and adopted in 2005 ( UNGA Resolution 60/147, 2005), shortly after the Commission began its work. It is reasonable to assume that a similar principle will apply to Russia: an aggressor state cannot assert its own claims or demands in the process of determining fair compensation for its victims, as this would contradict the very essence and spirit of law and justice.

The operational specifics of the Commission were gradually defined by the Governing Council through its decisions on matters within the Council’s competence. According to Decision No. 7 issued by the Governing Council of the UNCC, “direct loss” refers to any loss incurred as a result of:

a) Military operations or the threat of military action by either party between 2 August 1990 and 2 March 1991;
b) The departure of individuals from, or their inability to leave, Iraq or Kuwait during that period;
c) Actions taken by Iraqi government officials or agents in connection with the invasion or occupation;
d) The collapse of civil order in Kuwait or Iraq during the same timeframe; or
e) Hostage-taking or other forms of unlawful detention ( UNCC Governing Council Decision No. 7, 1992a, para. 6).

Building on this definition of direct loss, the Governing Council concluded that compensation could be granted for any direct loss, damage, or injury suffered by governments or international organisations as a consequence of Iraq’s unlawful invasion and occupation of Kuwait ( UNCC Governing Council Decision No. 7, 1992a, para. 34). Consequently, Iraq was obliged to compensate for any damage and losses caused by its military actions, even in the absence of proven violations of international humanitarian law, and even in cases where such damage or losses were caused by coalition forces.

The claims were classified into six categories: A, B, C, and D for individual claimants; category E for corporate entities; and category F for governments and international organisations. Category F included claims for environmental damage. A similar principle can be observed in the International Registry of Damage established under the efforts of the Council of Europe for compensating damages inflicted on Ukraine as a result of Russia’s invasion. The operational procedures of the compensation commission for Ukraine, including the categories of claims registered in the Registry of Damage, will be analysed in detail in subsequent studies.

The principle of claim submission to the Commission was based on state intermediation, depriving victims of the ability to submit claims directly to the Commission. Only the state had the right to seek compensation on behalf of its citizens and legal entities. In turn, the state collected claims centrally from victims to ensure their efficient and timely consideration by the Commission. This approach was necessary due to the high volume of individual claims, which required systematic processing.

It is also important to note the uneven temporal processing of claims across different categories. Analysis of available Commission documents and scholarly literature indicates that claims were reviewed according to priority, determined in part by the volume of submissions. For example, the Commission began reviewing claims related to environmental damage nearly ten years after the adoption of Resolution 687, which established the Commission, reflecting the low priority of this category in the overall process. Nevertheless, the unprecedented inclusion of environmental damage as a compensable category of conflict-related harm—caused by armed action or aggression—is precisely what has drawn significant attention to this Commission and underscores its undeniable contribution to the practice of international compensation commissions, being the first instance in which conflict-related environmental issues were addressed by a compensation commission.

As noted in the scholarly literature, a significant procedural and substantive limitation was the insufficient legal justification of Iraq’s state responsibility for its actions ( Cusato, 2019). Despite the obvious facts of aggression, crucial documents such as resolutions and decisions on holding a state accountable must provide clear justification of the principles and provisions of international law that have been violated to ensure the transparency and indisputability of the process. However, in its decisions, the UN Compensation Commission did not provide additional reasoning, referring solely to Resolution 687, in which the Security Council recognised Iraq’s responsibility for the invasion and occupation of Kuwait.

While Article 31 of the Provisional Rules for Claims Procedure provided that the Commissioners, despite the Commission’s quasi-judicial character, should apply not only the UNSC resolutions and Governing Council decisions but also other relevant rules of international law, this requirement was seldom explicitly relied upon ( UNCC Governing Council Decision No. 10, 1992b). According to classical deterrence theories, one of the ways to strengthen victim protection during armed conflict and to reinforce compliance with international obligations is by holding the offending state accountable. At the same time, however, to fully justify this objective, such responsibility must be clearly and legally substantiated.

It is also important to note the procedural features of evidence assessment in the work of the UN Compensation Commission. When state responsibility is adjudicated before a court or arbitral tribunal, evidence must establish a causal connection between the environmental harm and the specific conduct constituting a breach of international law, in line with applicable evidentiary and procedural standards ( Cusato, 2019).

The UNCC Governing Council adopted a notably flexible approach to both the admission and evaluation of evidence. It required that claims “be supported by documentary and other appropriate evidence sufficient to demonstrate the circumstances and the amount of the claimed loss” ( UNCC Governing Council Decision No. 7, 1992a, para. 37), yet it did not prescribe any specific standard of proof. Under the Provisional Rules for Claims Procedure established by the Governing Council, each Panel retained discretion to determine “the admissibility, relevance, materiality, and weight of any documents or other evidence submitted” ( UNCC Governing Council Decision No. 10, 1992b, art. 35). This flexibility in evidentiary standards underscores the sui generis—or exceptional—nature of the UNCC’s procedures ( Cusato, 2019).

Another challenging issue was the material assessment of compensation for damage that is difficult to monetise. In this context, Iraq even submitted objections, requesting limitations on the types of damage eligible for compensation. Iraq proposed that compensation should be paid only for damage that can be expressed in monetary terms. However, the Commission rejected this objection, emphasising that types of damage that are difficult to quantify financially cannot be excluded from the responsibility of the aggressor state.

As a result, the evaluation of such damage was based on the estimated cost of projects necessary to restore the affected resources, services, and infrastructure, or to develop new ones to replace those irreparably lost. This methodology was particularly applied in assessing compensation for environmental harm.

A closer analysis of the UNCC’s practice demonstrates that its departure from the strict application of the traditional rules of state responsibility—especially concerning causation and reparation—was essential to enable compensation for certain categories of conflict-related damage. This flexibility was largely made possible by the UNCC’s unique institutional character, which, unlike judicial bodies, was not bound by rigid procedural or evidentiary requirements, as well as by its distinct political and historical context.

The success of the UNCC was also due to Iraq’s acknowledgment of its responsibility for the damage caused to Kuwait, as codified in Resolution 687. According to the Basic Principles and Guidelines, the recognition by the aggressor state of its responsibility for harm is a crucial element in restoring the rights of victims ( UNGA Resolution 60/147, 2005). This acknowledgment enabled progress not only in awarding compensation but also in ensuring its enforcement. It is not sufficient to merely mandate compensation; the execution of this obligation must also be guaranteed. Just as in a classical judicial process, the realisation of the right to a fair trial does not end with the court hearing and the final judgment, so too the right to compensation reaches its logical conclusion only with the implementation of the decision by the authorised body and the actual receipt of the awarded compensation.

Overall, the work of the UNCC stands as a successful example of a specialised quasi-judicial body, demonstrating its legal efficacy. After 31 years of operation, as of January 2022, when the last payment for pending claims was completed, a total of $52.4 billion in compensation had been fully disbursed and distributed among 1.5 million claimants ( United Nations Compensation Commission, 2023). At its final session, the Governing Council adopted Final Decision No. 277 (2022), officially confirming that the Government of Iraq had fully complied with its international obligations by compensating all claimants who had been awarded reparations for losses and damages arising from Iraq’s invasion of Kuwait ( UNCC Governing Council Decision No. 277, 2022).

### 3.2 The compensation commission in Kosovo

The next example highlighted in this study is Kosovo. This state has been navigating the path of compensation for over 25 years—since the end of the conflict in 1999 up to the present. Such long-term experience can provide valuable insights in the search for an effective model for compensating damages caused by the war in Ukraine.

As noted in the OSCE 2020 report on Kosovo’s experience with implementing a mass-claims mechanism for property rights violations, although the conflict in Kosovo ended two decades ago, many property disputes remain unresolved today ( OSCE, 2020). The report rightly emphasises that one of the phenomena caused by armed conflict or acts of aggression is the mass displacement of persons from areas affected by hostilities, airstrikes, or other threats to life and health. In the post-conflict period, one of the greatest challenges is the return of previously displaced persons. However, many encounter the problem of having nowhere to return. The inadequate protection of displaced persons’ property rights remains a major barrier to their return. Resolving outstanding conflict-related property claims is a crucial prerequisite for establishing and sustaining stability in any post-conflict society, including Kosovo.

This underscores the importance of a methodical and systematic policy, accompanied by clear and specific legal regulation, to be implemented by responsible state authorities. After the 1999 conflict, such a role was assumed by the United Nations Interim Administration Mission in Kosovo (UNMIK), which supported and legitimised Kosovo’s governance in the temporary absence of local institutions. UNMIK was granted these powers under UN Security Council Resolution 1244 (1999), maintaining its mandate until local authorities were capable of assuming such responsibilities ( UN Security Council Resolution 1244(1999), 1999). To address claims and disputes related to property losses, the Housing and Property Claims Commission (HPCC) was established as a quasi-judicial body authorised to resolve private, non-commercial disputes concerning residential property ( UNMIK Regulation 1999/23, 1999). In addition, the Housing and Property Directorate was created as part of the compensation mechanism, responsible for the administrative support of the claims process. Interestingly, under the initial 1999 framework establishing the Commission, there were no appeal mechanisms, and Commission decisions could not be reviewed by any other judicial or administrative authorities in Kosovo. This situation changed somewhat in 2000, when the regulations were updated to introduce a procedure allowing a claimant or any interested party to request a review of the Commission’s decision—a step that strengthened the guarantee of fair compensation.

Similar to the UNCC established to compensate for damages caused by Iraq’s invasion of Kuwait, the HPCC’s jurisdiction was organised by categories: discrimination (Category A), informal transactions (Category B), and illegal occupation (Category C). Compared with the Compensation Commission created by the Council of Europe for Ukraine, the number of compensable categories in Kosovo was quite limited. Another distinctive feature of this categorisation is that it was based on the cause of the damage rather than the type of damage, reflecting the narrow specialisation of the Commission.

Damage caused by discrimination referred to losses suffered by individuals who were deprived of ownership, use, or possession of their residential property after 23 March 1989, as a result of discriminatory laws recognised as such both in content and intent. The Commission’s primary role was to determine whether the claimant’s rights were valid at that time and, if so, whether they had been nullified by discriminatory measures.

The second category included claims by individuals who conducted informal property transactions after the same date, based on the free will of the parties. This category was not strictly about compensation; rather, it concerned recognition of property rights by the Commission if it was established that the claimant had indeed acquired ownership through such informal agreements. Challenges in the property sector were further compounded by the widespread inaccuracy or complete absence of property records. Prior to 1999, numerous unofficial and informal property transfers occurred without registration, leaving records incomplete and unreliable. The situation worsened during the conflict, when many records were destroyed and others were taken by the authorities of the Federal Republic of Yugoslavia as they withdrawal in 1999— resulting in the majority of cadastral documents being transferred to Serbia ( Wühler, 2023; OSCE, 2020).

Finally, the third category included claims from individuals deprived of ownership, use, or possession due to illegal occupation. As noted in the OSCE report, claims in this category accounted for 93% of all claims received by the Commission during its operation, the majority of which were filed by displaced persons ( OSCE, 2020, p. 7).

The work of this Commission represented the first stage of damage compensation, while simultaneously signaling the priorities of international partners and the nascent state. The initial focus was on compensating losses to residential property in order to facilitate the return of the population, which was essential for sustaining the foundations of the newly formed state. Moreover, Article 156 of the Kosovo Constitution was later introduced, emphasising the goal of returning refugees and internally displaced persons and providing all necessary assistance to restore their property ( Constitution of the Republic of Kosovo, 2008).

Only after addressing the main body of claims for residential property compensation did the next step take place in 2006: the transformation of the Commission and Directorate into a new compensation body called the Kosovo Property Agency (KPA). This agency consisted of three components: an executive secretariat, a supervisory board, and the Kosovo Property Claims Commission (KPCC), a quasi-judicial body directly responsible for reviewing and deciding on claims. The KPCC’s powers were also expanded to receive, register, and assist courts in handling claims related to agricultural and commercial properties. Unlike claims concerning residential property losses, these claims pertained directly to damage caused by conflict-related events ( Wühler, 2023). Through its case law (i.e. Doğan and Others v Turkey (2004)) the ECtHR has established that states have an obligation to ensure displaced persons have secure access to their property, regardless of whether the state itself caused the conditions leading to displacement ( OSCE, 2020, p. 28).

The Executive Secretariat primarily concentrated on outreach initiatives and the collection and registration of claims. Once claims were registered, their processing involved several key steps: publishing and notifying claims, verifying submitted documentation, gathering additional evidence and conducting interviews, preparing written submissions for referral to the Commission, and presenting the claims for adjudication.

The Secretariat was headed by a Director and a Deputy Director, both appointed by the Kosovo Assembly following nomination by the Prime Minister. The Commission itself consisted of two international members and one local member. Initially, under UNMIK Regulation 2006/50, the international members were appointed by the Special Representative of the UN Secretary-General, who also designated one of them as Chairperson. However, this procedure was later amended by Law No. 03/L-079, which revised Article 10 of the Regulation. According to the new provisions, members of the KPCC were to be appointed by the Kosovo Assembly upon nomination by the President of the Kosovo Supreme Court ( Wühler, 2023).

A five-member Supervisory Board—composed of three international and two local members—was tasked with overseeing the work of the KPA, providing administrative supervision, strategic direction, and policy guidance. Over time, the Board’s composition and appointment procedures were modified through legal amendments. Although the Kosovo Assembly was responsible for appointing the national members, delays in the process led to the temporary establishment of a Stakeholders Committee in place of the Board. For a significant portion of its operation, the Board functioned without the participation of national members.

Thus, analysing the procedure for compensating damages caused by the 1998-1999 conflict in Kosovo, one can conclude that it was multi-staged and gradual. Additionally, both national and international expertise were actively involved in the functioning of the compensation mechanism, enhancing its transparency and effectiveness. Thanks to this balanced approach, the KPA and KPCC adapted mass claims methodologies originally developed in other mechanisms, drawing on the prior experience of their international members. Their approach involved grouping claims with similar legal and evidentiary characteristics, leveraging information technology and databases, standardizing workflows, and verifying claims in cases where claimants lacked access to essential evidence. This work was carried out with professionalism by a diverse team of national and international staff, who successfully navigated ethnic divisions and a politically sensitive environment.

Compensation cannot be rapid, especially in the context of armed conflicts and wars, as the damage is often multifaceted and widespread, necessitating a deliberate and carefully designed process, rather than one driven by hasty or populist decisions.

Equally important is adherence to due process. During the review of a claim regarding compensation for specific property (or other violated rights), the interests of all concerned parties must be considered. All persons affected by the matter under consideration should be involved—or at least informed—about the proceedings. For instance, in Kosovo, the Housing and Property Directorate was required to take all reasonable measures to notify both the resident of the claimed property and any other parties with a legal interest, informing them of the claim submission and their right to participate in the proceedings ( OSCE, 2020, p. 18). This approach ensures a comprehensive assessment of the circumstances, consideration of the rights and interests of all stakeholders, and the issuance of a fair decision.

As is currently the case in Ukraine, parallel judicial processes for conflict-related damages also took place in Kosovo, while the compensation commission continued its work. In such instances, different procedural systems coexist, which can duplicate administrative burdens and financial costs. When seeking fair compensation, a claimant may approach multiple forums without assessing the efficiency of each. Nonetheless, no right to access justice may be restricted. However, it may be reasonable to implement safeguards against parallel claims. For example, once a person submits a claim to the compensation commission, they should not be allowed to go to court until the commission has reviewed the claim and issued a decision.

By contrast, limiting access to courts is strongly discouraged. The right to judicial protection for violated rights is a fundamental human right, guaranteed by international instruments such as the Universal Declaration of Human Rights and the European Convention on Human Rights. The judiciary is one of the three essential branches of government in democratic states, distinguished constitutionally through the separation of powers. Justice is administered by independent and impartial courts, operating under the rule of law in line with European standards and ensuring the right to a fair trial for all. Courts are permanent structures, and their activities are characterised by consistency, ensured by uniform organisational and operational principles established at the legislative level.

Thus, filing claims in courts for compensation—as a permanently functioning institution that guarantees the comprehensive right to a fair trial—is a completely logical and expected step for victims in the absence of alternative means to obtain restitution. Experience from other countries that established compensation commissions shows that such commissions typically emerge in response to mass human rights violations, and their status is temporary. At the time of creating a compensation commission, thousands of compensation cases may already be pending in ordinary courts. Therefore, introducing a rule that would limit the right to approach a compensation commission if a person has filed a court claim during judicial proceedings would violate the principle of non-retroactivity of law and infringe the rights of those who resorted to courts due to the lack of alternative remedies.

In Kosovo, this issue was addressed as follows. The jurisdiction of ordinary courts over compensation claims extended only to categories of cases that were not within the competence of the Commission, or to matters not yet resolved by the Commission. In addition, the period for submitting claims to the KPA was limited, with the deadline for filing compensation claims set for December 2007. Anyone who missed this deadline could still submit a claim to an ordinary court ( Wühler, 2023). Furthermore, decisions of the KPCC could be appealed exclusively to the Supreme Court of Kosovo, establishing the Supreme Court’s exclusive jurisdiction in this category and rendering these cases non-justiciable in ordinary courts.

According to 2016 data, out of 41,849 decisions issued by the KPCC, a total of 1,293 appeals (3.09%) were filed with the Supreme Court of Kosovo. Of these appeals, 210 were upheld, while 663 were dismissed, thereby confirming the original KPCC decisions ( OSCE, 2020, p. 25). This statistic demonstrates the reduced workload for the judicial system and the effectiveness of the measures taken. However, despite the clear jurisdictional separation, the lack of proper coordination led Kosovo courts to open proceedings on the same claims under the jurisdiction of the compensation mechanism, resulting in parallel rulings and inefficient resolution of claimants’ disputes under legal uncertainty.

Reviewing a claim for compensation is only part of the right to fair restitution, which must be realised through direct enforcement of the Commission’s or quasi-judicial body’s decision. Therefore, a clear and effective enforcement procedure is of critical importance. To this end, at various stages of the compensation mechanism, specialised bodies were responsible for implementing the decisions of compensation commissions. During the KPCC’s existence, the KPA Executive Secretariat was responsible for enforcing its decisions. Enforcement measures included, for example, evicting occupants from illegally occupied property and demolishing unlawfully constructed buildings. However, few claimants made use of these remedies, as they were widely perceived as merely causing delays in the restitution process. Data from KPA annual reports, along with interviews with relevant institutions, indicated that no demolitions had occurred to date ( OSCE, 2020, p. 25). However, an obvious problem that led to the low utilisation of this enforcement mechanism was the insufficient budgetary resources to finance the procedure. This same problem affects victims seeking compensation for damages caused by Russia’s armed aggression against Ukraine. Clearly, the lack of funds that prevents the enforcement of decisions undermines the full realisation of the right to a fair trial—a fundamental prerequisite for all judicial protection. The implementation of court decisions is an essential component of this right, as confirmed by the European Court of Human Rights (ECtHR), which emphasises that an effective legal remedy must not remain merely theoretical but must also be applied in practice ( Hornsby v Greece, 1997, para. 41). It should be noted that non-enforcement of court decisions—in the broad sense of the term, which includes compensation commissions as quasi-judicial bodies—undermines the rule of law, constitutes a violation of fundamental human rights, and calls into question the capacity of responsible state authorities and international institutions to provide an effective compensation mechanism, and therefore to ensure the realisation and protection of human rights. Such violations cannot be justified by a lack of financial resources.

### 3.3 Compensation commission in Bosnia and Herzegovina

The Bosnian example of a compensation mechanism was not as successful as the mechanism that operated in Kosovo. The 1992–1995 Bosnian conflict involved severe and well-documented human rights violations, including killings, torture, rape, and mass displacement driven by ethnic cleansing. By the end of the war, half of Bosnia’s population of four million had been displaced—one million abroad and one million internally. Ethnic communities that had once coexisted were forcibly separated, and many homes were destroyed or reassigned, effectively preventing the return of their original inhabitants ( Williams, 2006).

The Commission for Real Property Claims (CRPC) was established under the Dayton Peace Agreement, which brought an end to the Bosnian War ( General Framework Agreement for Peace in Bosnia and Herzegovina, 1995). Its creation reflected the parties’ recognition that a just and lasting peace could not be achieved without addressing the property rights of displaced persons and refugees and taking measures to restore their violated rights. Accordingly, Article 1 of Annex 7 of the Agreement affirms the right of refugees and displaced persons to freely return to their homes and to receive compensation.

Article 1 establishes that all refugees and displaced persons are entitled to return freely to their homes of origin. They have the right to recover property lost during the hostilities since 1991 or, if recovery is not possible, to receive compensation. Facilitating the early return of displaced persons is emphasised as a key objective in resolving the conflict in Bosnia and Herzegovina ( General Framework Agreement for Peace in Bosnia and Herzegovina, 1995, annex 7, ch. 1, art. 1).

Chapter II of Annex 7 to the Dayton Peace Agreement established a dedicated institution, the Commission for Real Property Claims of Displaced Persons and Refugees (the Commission), to address the complex and sensitive issues related to real property. The Commission was tasked with receiving and adjudicating claims for real property in Bosnia and Herzegovina where the property had not been voluntarily sold or otherwise transferred since 1 April 1992, and where the claimant did not currently possess the property. Claims could seek either the return of the property or compensation in lieu of return ( Van Houtte, 1999). Within eight years of the mass claims settlement, the CRPC had verified ownership rights to approximately 320,000 dwellings and established a legal framework for their enforcement ( Van Houtte, 2025).

The formation of the compensation commission in Bosnia differed from the previously discussed cases. One most notable feature was the involvement of the European Court of Human Rights (ECHR) in the Commission’s activities. The Commission consisted of three international members and six national members, ensuring the necessary balance of representation and theoretically promoting independence and transparency in its decisions.

The appointment of the international members was entrusted to the President of the ECHR, which is unusual. The Dayton Peace Agreement does not explain why this function was specifically assigned to the President of the ECHR or the rationale behind this distribution of powers. The President’s mandate includes representing the Court in relations with the Council of Europe bodies as well as with the political and judicial authorities of the member states of the European Convention on Human Rights. The choice was likely justified by the supranational character of the ECHR’s jurisdiction, trust in the Court as an institution dedicated to the protection of human rights, and the impartiality of the President as its highest representative ( Viljanen, 2011).

The Commission also comprised six members from Bosnia and Herzegovina. The Federation of Bosnia and Herzegovina appointed four members—two serving three-year terms and two serving four-year terms—while the Republika Srpska appointed two members, one for a three-year term and the other for a four-year term. International members were appointed for five-year terms. The Agreement imposed no restrictions on the number of terms a member could serve. All appointments were to be made within 90 days of the Agreement’s entry into force ( General Framework Agreement for Peace in Bosnia and Herzegovina, 1995, annex 7, ch. 2, art. 9). An interesting aspect is the unequal duration of the members’ terms, which is clearly asymmetrical.

Although the Dayton Agreement allow the Commission to operate in panels, the proposed structure—three panels each composed of one international and two national members—proved unworkable, as it excluded representation from the third ethnic group in each panel ( Van Houtte, 1999). For this reason, it was decided that, instead of operating through panels, the Commission would establish two working groups, whose work focused on legal and operational matters, respectively. In addition, the national members held regular meetings between the sessions of the working groups, during which they discussed specific claims from affected individuals, draft proposals for their resolution, and other matters critical to the commission’s activities.

The Commission can be characterised as a higher and non-appealable instance. According to the procedure established by the Agreement, an individual whose property rights were violated must first apply to the municipal authorities, which have the power to issue a decision aimed at restoring the violated rights. However, if a pre-war occupant of an apartment or the owner of a house is dissatisfied with domestic proceedings or municipal decisions, they may submit a claim to the Commission for return of their property ( Van Houtte, 1999). Filing such a claim suspends all ongoing proceedings, including the enforcement of prior decisions, until the Commission issues its final ruling, which is binding ( General Framework Agreement for Peace in Bosnia and Herzegovina, 1995, annex 7, ch. 2, art. 12). For displaced persons and refugees who could not secure a directive from local authorities, the Commission effectively served as the primary mechanism for obtaining restitution.

The categories of cases heard by the Commission were clearly defined and, in substance, were similar to the categories addressed by the compensation commission in Kosovo, as analysed earlier in this article. Cases within the Commission’s jurisdiction were divided into four types: recognition of ownership or possession rights; termination of agreements unlawfully concluded during the war; return to previously abandoned residential property; issues related to property reconstruction, since compensation under this last category could only be granted in cases where reconstruction was conducted to restore the legal owner’s or possessor’s ability to reside in the reconstructed property ( General Framework Agreement for Peace in Bosnia and Herzegovina, 1995, annex 7, ch. 2, art. 11). The largest number of claims fell under the first category.

Claims could seek either the physical return of property or fair compensation as an alternative. By accepting compensation, claimants relinquished both their ownership rights and their right to reclaim the property. However, in practice, the Commission did not issue decisions directly on the return of property or on the provision of fair compensation, for reasons that will be discussed below. Its work was limited to recognising the violated ownership right over specific property, leaving the owner free to exercise that right in the manner they deemed appropriate.

The Commission operated on the principle of territoriality. Its main office was located in Sarajevo, and Annex 7 of the Agreement allowed the establishment of subordinate offices in other locations where necessary. This approach enhanced accessibility for affected persons, created a sense of direct dialogue between claimants and the Commission, and encouraged their active participation in the claims process.

The creation of regional offices helped streamline the collection and registration of claims related to violated rights, as well as the documentation of supporting evidence. The Commission established offices not only within Bosnia and Herzegovina but also in countries hosting significant populations of Bosnian displaced persons, including Croatia, Serbia, Montenegro, Denmark, Germany, the Netherlands, and Norway ( Van Houtte, 2025).

The Commission faced a number of challenges. Article 9 of Annex 7 stipulated that compensation for property in cases where owners refused to return was to be determined based on a fixed valuation of the property as of 1 January 1992, i.e., before the outbreak of the Bosnian War. In reality, however, it was practically impossible to carry out such valuations for most properties due to the damage and destruction caused by the four-year conflict.

Problems also arose with the form of compensation, which was to be paid either in cash or in the form of compensation bonds. However, there were no realistic prospects of establishing a real estate market that would allow these bonds to facilitate property exchange or acquisition. Although the Dayton Agreement seemed to envision the CRPC playing a central role in such a market, it lacked the resources and time to do so. None of the international bodies involved took on this sensitive task, and the existing property sector was dominated by criminal networks. Without a functioning real estate market, issuing compensation bonds was deemed irresponsible due to their uncertain value and limited practical utility ( Van Houtte, 2025). In this case, it is evident that the Bosnian compensation mechanism, though formally established and regulated at the normative level, clashed with realities it could not withstand or overcome. This demonstrates that innovative external solutions will have no effectiveness or practical implementation if the domestic state system and legislation are not prepared for them. If a right enshrined in an international or national legal act and guaranteed by the state lacks a clear mechanism for its implementation, that right is at risk of being negatively violated.

Monetary compensation also proved problematic to implement. According to the Agreement, payments were to be made from a specially established Property Fund, administered by the Commission, with funds held at the Central Bank of Bosnia and Herzegovina. The Fund was to be financed through contributions from the Republic and its two constitutional entities, as well as from third states, international, and non-governmental organisations. Its revenues would support compensation for property claims, especially as many claimants did not wish to return and pre-war property values had to be considered. However, the Fund never received any money. With no financial resources, the CRPC opted not to provide compensation, leaving both core elements of its mandate—return and compensation—unfulfilled.

Unlike the compensation commission in Kosovo, the Bosnian Commission did not limit the jurisdiction of courts over claims for restitution or the restoration of violated rights. The Commission did not have exclusive authority over this category of cases, so local general courts and executive authorities retained their powers to consider such claims. Nevertheless, the Commission served as an alternative whenever national institutions were unable to deliver fair and adequate decisions. However, this approach led to inefficient distribution of workload among available remedies and contributed to parallel proceedings, which was partly caused by the lack of a clear division of competences between national authorities and the Commission as an international compensation mechanism.

It should be emphasised, however, that Commission decisions were final and unappealable. No court had jurisdiction to review, amend, or overturn its decisions. Even the Constitutional Court of Bosnia, in the case Siniša Stanisavljević v. CRPC ( Case AP-2264/07, 2008), deemed an appeal against a Commission decision inadmissible, stating that it was not competent to review decisions made by a body established by the Dayton Agreement and endowed with an international mandate.

The CRPC functioned as a fast-track mechanism designed to reduce the workload of domestic courts by resolving property claims through simplified administrative procedures without holding hearings. It did not have the time, resources, or mandate to evaluate the legality of wartime transactions, leaving such disputes to domestic courts. Investigating possible duress in property transfers would have delayed its work, so these cases were excluded from its scope. Facing hundreds of thousands of claims within a limited timeframe, the Commission streamlined its processes as much as possible, making decisions solely based on the submitted claim forms and supporting evidence provided by claimants or independently collected by the Commission. Over the course of its operation, the CRPC registered a total of 240,233 claims concerning 319,220 properties ( Van Houtte, 2025).

## 4. Conclusions and recommendations

The previous sections of this study reveal different approaches in establishing special ad hoc compensation commissions regarding the rights of victims to apply to quasi-judicial bodies. Essentially, this difference reflects a distinct approach to the right of access to justice. Some commissions practiced centralised collection of claims from victims through the state affected by aggression, while others ensured the right of direct individual application for compensation.

A logical question arises: which approach should be adopted for the Compensation Commission for Ukraine? It is entirely justified and fair to adopt a mechanism of direct submission of claims by all victims to the Commission, as this is based on the individual procedural right of each person to seek compensation for harm caused specifically to them. Accordingly, the right to submit a claim must correspond to this individual character.

This view is reinforced by established ICC practice, which recognises the individual right to reparation as a fundamental human right. This right is widely acknowledged across universal and regional human rights instruments, as well as in international guidelines and declarations addressing justice for victims, the protection of children in armed conflict, and the social reintegration of former child soldiers. Such cross-disciplinary developments underscore the broad acceptance and importance of ensuring reparations for individuals ( Case ICC-01/04-01/06, 2012, para. 185).

However, it is important to emphasise that the individual right to reparation can be recognised under international law only when a person has the right to submit a claim through an international procedure, such as ad hoc compensation processes, and when such procedure and its rules are provided for in an international legal instrument ( Correa et al., 2020).

The study examined and analysed various types of harm, including damage caused to displaced persons through internal and external displacement, environmental harm, and harm to property rights. It also identified and analysed the features of the establishment of compensation commissions specifically focused on these human rights violations and the restoration of the infringed rights.

The analysis of the activities of the compensation commissions considered in this study allows for several important conclusions, which are relevant for the development and implementation of a future compensation mechanism in Ukraine.

First, a common feature of all the commissions examined is the presence of a clear delineation of substantive jurisdiction. Both the types of harm that could be compensated and the temporal limits or specific events within which such harm occurred were clearly defined. Only damage caused during the specified period or as a result of the identified events was subject to compensation. This approach ensured the objectivity and reasonableness of claim consideration.

Second, in none of the cases analysed was compensation paid quickly. The process proved to be lengthy and complex not only legally but also politically. This indicates that in developing a compensation mechanism for Ukraine, one should not expect rapid decisions or immediate payouts – it is important to realistically assess the prospects of its operation.

Furthermore, the effectiveness of the compensation process largely depends on the clarity, transparency, and predictability of the procedure. Only a well-founded and consistent procedure, strictly followed by all parties, can ensure the legitimacy and recognition of the decisions of the compensation body. Otherwise, there is a risk of losing trust in the entire mechanism.

Special attention should be paid to the issue of alternative means of compensation and classical judicial proceedings. Considering these mechanisms and their relationship within the overall system can help avoid duplicate claims, parallel proceedings, and contribute to resource efficiency. Achieving this is possible through a clear delineation of jurisdictions (substantive, appellate, territorial, etc.) among the bodies where individuals may seek compensation, while adhering to the principle of legal certainty.

Finally, a particularly unresolved problem remains the enforcement of decisions by compensation bodies. Without an adequate enforcement mechanism, the right to compensation remains declarative, and the realisation of the right to a fair trial is incomplete. Experience from other mechanisms shows that this problem is systemic and current and requires further detailed study.

This article aimed to explore the experience of creating and operating compensation commissions worldwide. The experience of previous compensation commissions provides valuable guidance for Ukraine, but it requires careful consideration of legal, institutional, and practical aspects, as well as adaptation to the national context. Future research plans include a more in-depth analysis of the procedural aspects of reviewing claims for damage caused by armed aggression and other conflicts, drawing on both international and Ukrainian experience.

## Ethical considerations

Ethical policy requirements have been met. The article does not involve human participants or animals and does not include results from research involving them.

## Disclosure

In preparing this manuscript, I utilised ChatGPT (free version) exclusively to assist with translation and to ensure the text’s lexical and grammatical accuracy, since English is not my native language. No substantive content, analysis, or conclusions were generated by the tool.

**Corresponding author** is solely responsible for the manuscript preparing (Conceptualization, Investigation, Methodology, Writing – Original Draft Preparation).

## Data Availability

This study did not involve the generation or analysis of any datasets. All data supporting the findings of this article consist of bibliographic references, which are fully provided in the References section.
