# Supplementary material for: International Experience of Damages Compensation in Armed Conflicts: Lessons for Ukraine
Source: F1000Res. 2025 Nov 13;14:1247. [Version 1] doi: 10.12688/f1000research.171894.1 (PMC12645091; doi:10.12688/f1000research.171894.1)
Supplement: Peer review history

# Reviewer report: Reviewer response for version 1

**DOI:** 10.5256/f1000research.189558.r433054

**Published:** 2025-11-24

**Authors:** Qerim Qerimi

**Affiliations:** 1 Law, University of Antwerp, Antwerp, Belgium; 2 University of Prishtina, Prishtina, Kosovo (Serbia and Montenegro)

I think this is timely contribution. The article draws on on other pertinent experiences, to identify the best modality for addressing reparations in a post-war Ukraine. Methods are appropriate and literature used relevant. The results are clear and concrete, contributing to existing understanding and debate about the procedure and process of how to arrange or design the institutional structures for effectively addressing the right to just compensation.

Is the case presented with sufficient detail to be useful for teaching or other practitioners?

Yes

Is the work clearly and accurately presented and does it cite the current literature?

Yes

If applicable, is the statistical analysis and its interpretation appropriate?

Yes

Are all the source data underlying the results available to ensure full reproducibility?

Yes

Are the conclusions drawn adequately supported by the results?

Yes

Is the background of the case’s history and progression described in sufficient detail?

Yes

Reviewer Expertise:

International Law, Human Rights, Rule of Law

I confirm that I have read this submission and believe that I have an appropriate level of expertise to confirm that it is of an acceptable scientific standard.

# Reviewer report: Reviewer response for version 1

**DOI:** 10.5256/f1000research.189558.r433052

**Published:** 2025-11-20

**Authors:** Maya Khater

**Affiliations:** 1 Abu Dhabi University, Abu Dhabi, United Arab Emirates

I recommend accepting this article for the following reasons:

 The article addresses a timely and highly normative issue: reparations for victims of armed conflict, with a clear focus on the implications for Ukraine following the 2022 invasion. It provides a robust comparative analysis of three major reparations mechanisms (the UN Compensation Commission, Kosovo, and Bosnia and Herzegovina), carefully selected and clearly justified in their relevance to the Ukrainian context. The study is empirically grounded, drawing on UN and Council of Europe instruments, OSCE reports, NGO documents, and academic research, thus strengthening the credibility of its findings. The paper successfully connects institutional design to the broader issue of access to justice and the individual's right to reparation. The conclusions are highly relevant to policy and offer concrete lessons for developing a future reparations mechanism for Ukraine, particularly regarding jurisdictional clarity, transparency, and implementation challenges. The study is logically structured, and the argument is generally easy to understand.

 Suggestions for Improvement

 It would be helpful to clearly formulate one or two key research questions at the end of the introduction, along with a brief statement of the article's original contribution.

 Please shorten some of the historical and institutional descriptions, particularly in the lengthy sections relating to the UN Compensation Commission and Kosovo. This will allow more space to highlight the normative framework for the right to compensation (the UN Basic Principles and regional human rights case law) or to provide an analytical comparison with Ukraine.

 The bibliography is generally sufficient, but it could be enriched with recent studies on reparations and collective claims procedures in armed conflicts, as well as comparative works on environmental damage and reparations. In this regard, I recommend referring to the following recent article, which addresses state responsibility and compensation for victims of the Syrian war, and which may enrich your comparative discussion of compensation mechanisms:

 Khater, M., Alhowaish, Y., Chami, Y., El Arbi Aoka, M., & Hammad, H. “Legal Responsibility of Iran for Compensating Victims of the Syrian War”, Access to Justice in Eastern Europe, 8(4), 1–27, 2025. https://doi.org/10.33327/AJEE-18-8.4-a000126

 Best wishes,

 Maya

Is the case presented with sufficient detail to be useful for teaching or other practitioners?

Yes

Is the work clearly and accurately presented and does it cite the current literature?

Yes

If applicable, is the statistical analysis and its interpretation appropriate?

Not applicable

Are all the source data underlying the results available to ensure full reproducibility?

Yes

Are the conclusions drawn adequately supported by the results?

Yes

Is the background of the case’s history and progression described in sufficient detail?

Yes

Reviewer Expertise:

Public International Law

I confirm that I have read this submission and believe that I have an appropriate level of expertise to confirm that it is of an acceptable scientific standard.
